# The Influence of Context on Implementation and Improvement: Protocol for a Mixed Methods, Secondary Analyses Study

**DOI:** 10.2196/40611

**Published:** 2022-09-15

**Authors:** Carole Estabrooks, Yuting Song, Ruth Anderson, Anna Beeber, Whitney Berta, Stephanie Chamberlain, Greta Cummings, Yinfei Duan, Leslie Hayduk, Matthias Hoben, Alba Iaconi, Holly Lanham, Janelle Perez, Jing Wang, Peter Norton

**Affiliations:** 1 Faculty of Nursing University of Alberta Edmonton, AB Canada; 2 School of Nursing Qingdao University Qingdao China; 3 School of Nursing University of North Carolina at Chapel Hill Chapel Hill, NC United States; 4 School of Nursing Johns Hopkins University Baltimore, MD United States; 5 Institute of Health Policy, Management and Evaluation University of Toronto Toronto, ON Canada; 6 Department of Medicine University of Texas Health Sciences Center San Antonio San Antonio, TX United States; 7 Department of Family Medicine University of Calgary Calgary, AB Canada

**Keywords:** organizational context, facilitation, PARIHS framework, Promoting Action on Research Implementation in Health Services, implementation science

## Abstract

**Background:**

Caring for the well-being of older adults is one of the greatest challenges in modern societies. Improving the quality of care and life for older adults and the work lives of their care providers calls for effective knowledge translation of evidence-based best practices.

**Objective:**

This study’s purpose is to contribute to knowledge translation by better understanding the roles of organizational context (workplace environment) and facilitation (process or role) in implementation and improvement success. Our study has 2 goals: (1) to advance knowledge translation science by further developing and testing the Promoting Action on Research Implementation in Health Services framework (which outlines how implementation relies on the interplay of context, facilitation, and evidence) and (2) to advance research by optimizing implementation success via tailoring of modifiable elements of organizational context and facilitation.

**Methods:**

This is secondary analyses of 15 years of longitudinal data from the Translating Research in Elder Care (TREC) program’s multiple data sources. This research is ongoing in long-term care (LTC) homes in western Canada. TREC data include the following: 5 waves of survey collection, 2 clinical trials, and regular ongoing outcome data for LTC residents. We will use a sequential exploratory and confirmatory mixed methods design. We will analyze qualitative and quantitative data holdings in an iterative process: (1) comprehensive reanalysis of qualitative data to derive hypotheses, (2) quantitative modeling to test hypotheses, and (3) action cycles to further refine and integrate qualitative and quantitative analyses. The research team includes 4 stakeholder panels: (1) system decision- and policy makers, (2) care home managers, (3) direct care staff, and (4) a citizen engagement group of people living with dementia and family members of LTC residents. A fifth group is our panel of external scientific advisors. Each panel will engage periodically, providing their perspectives on project direction and findings.

**Results:**

This study is funded by the Canadian Institutes of Health Research. Ethics approval was obtained from the University of Alberta (Pro00096541). The results of the secondary analyses are expected by the end of 2023.

**Conclusions:**

The project will advance knowledge translation science by deepening our understanding of the roles of context, the interactions between context and facilitation, and their influence on resident and staff quality outcomes. Importantly, findings will inform understanding of the mechanisms by which context and facilitation affect the success of implementation and offer insights into factors that influence the implementation success of interventions in nursing homes.

**International Registered Report Identifier (IRRID):**

DERR1-10.2196/40611

## Introduction

### Background

Concerns about quality in long-term care (LTC) homes are not new—the literature overflows with decades of calls to improve quality of care in LTC [1-4]. International [3,5], national [6-9], and provincial [10-12] reports highlight the suboptimal quality of LTC. Effective programs for improving quality of care in LTC homes are available, but implementation of these evidence-based programs has had inconsistent success across studies [1,13]. Implementation success is a proximal outcome that should lead to more distal improvement in outcomes for resident care quality or staff quality of work life (improvement success). One key outcome indicating implementation success in health care settings is the uptake of best practices (also called best practice use or research use) by health care workers such as physicians, regulated nurses, and unregulated staff. Researchers have noted a persistent lack of success in implementing evidence-based programs and cite a lack of understanding of the interrelating factors influencing implementation as a major knowledge gap [14,15].

### Theoretical Framing: the Promoting Action on Research Implementation in Health Services Framework

The Promoting Action on Research Implementation in Health Services (PARIHS) framework is widely used in implementation science [16-18] and offers a guide for implementing quality improvement programs in health care settings. This framework proposes that successful implementation of research evidence depends on the interplay of context, facilitation, and evidence [16-18]. In this study, we focus on two of the framework’s key elements: context and facilitation. They have critical roles in influencing implementation and improvement success—and outcomes for residents and care staff.

The PARIHS definition of context is highly general: the setting where a proposed change is to be implemented [17]. PARIHS developers initially conceptualized context as including culture, leadership, and evaluation (feedback of data to end users). Researchers using the framework now acknowledge more components of context, generally divided into inner context (immediate local setting, the organization) and outer context [19,20] (health system of organization and policy, social, regulatory, and political infrastructures) [16]. Context is highly modifiable using strategies to improve service quality and outcomes [21-23] and is therefore vital to improvement initiatives [24,25]. Research increasingly emphasizes the central role of organizational context in the success of both implementation and improvement initiatives, and its influence on workforce and resident outcomes in LTC homes [24,26-31]. Accordingly, researchers increasingly focus on organizational context (context within the workforce) as a core element of quality improvement initiatives [15,32] and consider the influence of context on implementation [16,33].

Elements of context (eg, leadership, social capital, decision-making autonomy, and communication) are associated with outcomes for direct care staff in LTC homes, such as job satisfaction, burnout, use of best practices, and tasks rushed or left undone [26,27,34-39]. Context elements also influence quality of life and care for LTC residents [28]. Residents in LTC homes with a more favorable context had significantly lower burdensome symptoms (eg, pain, shortness of breath, and urinary tract infection) and lower use of antipsychotics without a diagnosis of psychosis [28]. A review of qualitative studies found that poor context (culture that is not collaborative, is hierarchical, or has leadership that is poorly connected with realities faced by staff) negatively affected performance (eg, quality of care) in health care organizations [40].

However, despite decades of work on context, no coherent body of evidence clearly demonstrates context conditions for success [14,24,25]. Work from this research team has provided early evidence of the influence of context on LTC staff and resident outcomes [28], but further research is needed to deepen our understanding of these relationships. With these studies, we can begin to understand the complex causal mechanisms and the necessary and sufficient context conditions that produce particular outcomes for implementation and improvement success.

### Facilitation Influences Implementation and Improvement Success

The PARIHS framework considers a second component, facilitation, as both a role (eg, coach or educator) and a process (of enabling others) [41]. Emerging research highlights facilitation’s critical role in implementation and improvement initiatives [42-48]. A 2012 systematic review found that primary care settings supported by a facilitator were 2.76 times more likely to adopt evidence-based clinical guidelines [49]. In our recent empirical work, we successfully improved the involvement of LTC care aides in formal communications [29,50]. We also used targeted facilitation interventions, with external quality experts supporting LTC managers to change context-of-care units using goal setting theory [29,50].

Despite these insights, a lack of theoretical grounding is one factor that continues to limit our understanding of the effects of facilitation on implementation success [46,48]. Theoretical analysis suggested that varied effectiveness of facilitation is related to factors that influence organizational learning, because facilitation acts as a learning mechanism [48]. Optimizing facilitation (within a given context) may amplify organizational learning processes, making it easier to tailor facilitation to local context [47].

### Context Elements and Facilitation Interact to Influence Implementation Success

The PARIHS framework suggests that context factors and facilitation interact in complex ways, but neither the PARIHS developers nor other researchers have addressed directionality, addressed mechanisms of interactions, or actively and empirically assessed wider context factors (Y Duan et al, unpublished data, forthcoming) [41,51]. Some evidence points to facilitation as a key ingredient in implementation initiatives, but only preliminary work in small-scale qualitative studies addresses the influence of interactions between facilitation and context [52]. In previous work, we used cross-sectional data and demonstrated that the association of leadership with use of best practices by LTC care staff was moderated by clinical educators—an internal facilitator role in LTC homes [30]. Further research is warranted for a deeper understanding of how and under what conditions do context and facilitation interact and how these interactions affect resident and care staff health and well-being.

### Previous Work

Our integrated knowledge translation program, Translating Research in Elder Care [53] (TREC), was founded to focus on (1) contributing to knowledge translation or implementation science and (2) developing practical research-based solutions to improve LTC quality of care, life, and work life. Preceding this project, we (1) developed instruments that measure PARIHS constructs in LTC [54,55], (2) framed clinical trials drawing on PARIHS [29,56,57], and (3) demonstrated strong associations between organizational context and specific staff and LTC resident outcomes and between organizational context and implementation success [26-28,37,58].

### Study Purpose, Goals, and Aims

The purpose of this study is to contribute to knowledge translation science by better understanding the roles of organizational context and facilitation in implementation and improvement success. We have 2 broad goals: (1) to advance knowledge translation science by further developing and testing the PARIHS framework for research implementation and (2) to lay foundations to advance research by optimizing implementation success via effective tailoring of modifiable elements of organizational context and facilitation. Our specific study aims are to as follows: (1) to identify how context influences success of implementation and of quality improvement initiatives, (2) to identify and map context conditions in which facilitation affects outcomes for staff quality of work life, and (3) to identify and map context conditions in which facilitation affects resident outcomes.

We propose that improvements in care and better implementation success can be achieved by modifying elements of context, particularly inner context (immediate local setting and the organization), and through both optimal use of facilitation roles and enabling of facilitation processes [41,48,59].

## Methods

### Ethics Approval

Ethics approval for this study was obtained from the institutional review board of the University of Alberta (Pro00096541).

### Study Design

In these secondary analyses, we will apply convergent mixed methods and sequential mixed methods designs [60] to concurrently address all three of our aims.

### Overall Approach

The research team includes a multidisciplinary group of researchers, 4 panels of key stakeholders who are end users, and a fifth panel of external scientific experts: (1) system-level decision- and policy makers, (2) LTC home managers, (3) direct care staff working in LTC homes, (4) people living with dementia and their family members (citizen engagement), and (5) scientists with substantive knowledge of organizational context and learning, the PARIHS framework, context, facilitation, leadership, and implementation science.

**Figure 1:**
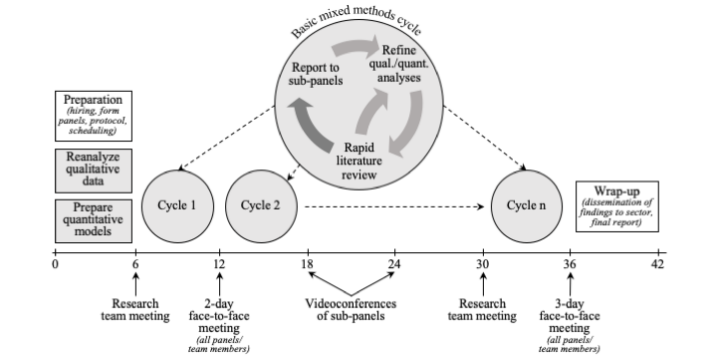
Project overview.

We will analyze TREC’s comprehensive data holdings (qualitative and quantitative data) in an iterative process (action cycles; see Figure 1): (1) comprehensive analysis of qualitative data to derive hypotheses on links between context factors and specific outcomes not yet explored, (2) analysis of quantitative data to test derived hypotheses, and (3) mixed methods action cycles to integrate qualitative and quantitative findings. Each action cycle will consist of data analysis, rapid literature reviews, and expert panel consultations and synthesis to further refine and integrate qualitative and quantitative analyses (Figure 1).

### Setting, Sample, and Data Holdings

#### Setting and Sample

The TREC program is situated in residential LTC in the 4 western Canadian provinces (British Columbia, Alberta, Saskatchewan, and Manitoba) where we maintain a cohort of 94 participating LTC homes. The cohort sample is a stratified (health region, owner-operator model, and bed size) random sample [61] of urban LTC homes in 5 health regions of participating provinces.

#### TREC Data Holdings

We have longitudinal data (Table 1) from 2 main sources: (1) TREC surveys (collected in 5 waves since 2007) of LTC homes, care units, and all levels of staff (regulated and unregulated nursing staff, allied health providers, specialists and educators, and managers) and (2) administrative data collected using the Resident Assessment Instrument – Minimum Dataset (RAI-MDS) 2.0 [62]. The RAI-MDS 2.0 is a routinely collected (quarterly, annually) and standard mandated assessment of clinical and functional outcomes for LTC residents [62]. Beyond these observational data from our ongoing cohort study, we have data from pilot studies, clinical trials, and case studies. All research data are housed in the Health Research Data Repository (University of Alberta), which provides virtual data access for team members within a highly secure environment. Data are extensively processed to exceed Canadian Institute for Health Information standards for RAI-MDS 2.0 data. Extensive quality assurance (during data collection and post data collection) is carried out with survey data, including assigning each resident to a single unit within a single LTC home [63].

**Table 1:** Overview of Translating Research in Elder Care (TREC) data holdings from 5 waves of data collection.

Data source				Participants, n								
				Wave 1 (June 2008 to July 2009)		Wave 2 (July 2009 to June 2010)		Wave 3 (September 2014 to May 2015)		Wave 4 (May 2017 to December 2017)		Wave 5 (September 2019 to March 2020)
**TREC survey**												
	Long-term care home		36		36		91		94		91	
	Care unit		103		103		336		339		324	
	**Care staff**											
		Care aides	1489		1506		4065		4158		3765	
		Nurses (registered nurses and licensed practical nurses)	277		308		767		927		931	
		Allied health professionals^a^	119		145		338		569		544	
		Specialists	24		21		57		80		59	
		Managers	55		69		168		193		199	
		Physicians^b^	9		16		0		0		0	
**Resident Assessment Instrument – Minimum Data Set 2.0**												
	Full assessments		5326		5087		13,956		12,290		9832	
	Quarterly assessments		12,195		11,453		19,467		19,240		14,238	
	Unique residents		5593		5549		14,139		13,852		13,158	
**Case studies^c^**												
	Interviews		70		0		0		0		0	
	Field notes		22		0		0		0		0	

^a^Allied health professionals surveyed include rehabilitation therapists (physical therapists and occupational therapies); clinical pharmacists; respiratory therapists; recreation therapists; social workers; dieticians; speech language pathologists; rehabilitation therapist assistants, attendants, and aides; and recreation therapist assistants, attendants, and aides.

^b^Physicians were not included in waves 3-5 because relatively few are regular participants in long-term care (LTC) delivery in our Canadian LTC system.

^c^Case studies include interview data with LTC direct care staff and management and administrative staff, and field notes from nonparticipation observation and document review.

### Clinical Microsystems

We are able to link TREC data from multiple sources at the level of the clinical microsystem (LTC resident care unit). Data are linked by assigning residents and staff to specific care units (clinical microsystems) to create longitudinal data sets at the care unit level within LTC homes. The clinical microsystem, a central concept in quality improvement science, is the level where care is organized and delivered [64,65] and where targeted strategies are most likely to improve quality [66-68]. An innovation in the TREC research program has been to link data at the LTC resident care unit level, including administrative data (eg, standard assessments of residents). We showed that the care unit is an appropriate level to introduce and test interventions, and that it can be characterized by core internal context constructs [26,27,29,56]. We demonstrated that measuring quality indicators at the LTC home level masks important variance between care units within LTC homes [54,69].

### Measures

#### Survey Data

Data captured in the TREC survey include (1) structural characteristics: LTC home surveys completed by the LTC home administrator and unit surveys completed by the unit care manager and (2) individual measures from managers, regulated nursing staff, allied health staff, and unregulated care aides (nursing assistants).

Examples of structural characteristics are size and the owner-operator model of LTC homes and type and staffing level of care units. Individual surveys are a suite of instruments and questions that capture demographic characteristics, best practice use, quality of work life, and organizational context. Quality of work life variables are captured using validated measures. They include burnout (Maslach Burnout Inventory) [70], physical and mental health (Short Form-8) [71], work engagement (Utrecht work engagement scale) [72], psychological empowerment (Psychological Empowerment Scale) [73], organizational citizenship behavior [74], job satisfaction (positively phrased version of Michigan Organizational Assessment Questionnaire Job Satisfaction Scale) [74], and responsive behaviors of residents toward staff [75].

Organizational context is captured using the Alberta Context Tool (ACT) [54,55]. We developed the ACT based on the PARIHS initial conceptualization of context, which includes culture, leadership, and evaluation (feedback of data to end users). We added constructs substantiated in health services literature [76], such as social capital, formal and informal interactions (2 communication concepts), organizational slack, and resources [55]. The ACT has measured context at the LTC home, unit, and group levels [27,28], but it is designed specifically for the clinical microsystem or resident care unit level [26-29]. It has had extensive psychometric assessments [54,55,77-79].

#### Resident Data

We have access to deidentified resident data from the RAI-MDS 2.0 for each of the 94 participating LTC homes. From our 5 large waves of data collection, we have over 500,000 resident data records. Further, we can link these data to external Continuing Care Reporting System data nationally (eg, Canadian Institute for Health Information [80]) and provincially (eg, Alberta Health [81]), and to other Canadian administrative databases (eg, Discharge Abstract Database [82]).

#### Clinical Trial Data

Table 2 provides an overview of data from our 2 completed clinical trials: Improving Nursing home care through Feedback On performance (INFORM) data [50,83,84] and Safer Care for Older Persons [in residential] Environments (SCOPE) [56,85,86]. The INFORM trial supported LTC home managers as they used our feedback from survey findings. INFORM included 143 care units from 58 LTC homes. It gathered quantitative data from surveys, reports, and workshop and rating evaluations, along with qualitative data from focus groups, interviews, and reports (process evaluation data).

The SCOPE trial examined the effects of empowering care aides to lead quality improvement strategies in their care unit. SCOPE included 408 participants from 45 LTC homes. It gathered quantitative data as rich survey data from individuals, teams, and leadership, and qualitative data from interviews, focus groups, and observation (process evaluation data).

**Table 2:** Overview of Improving Nursing home care through Feedback On perfoRMance and Safer Care for Older Persons [in residential] Environments trial data.

Data source			Value, n
**Improving Nursing home care through Feedback On perfoRMance**			
	Long-term care homes		58
	Care units		143
	**Surveys (participants)**		
		Fidelity checklist	278
		Workshop evaluation	355
		Report back slides	167
	Focus groups (units)		60
	Interviews (units)		11
	Engagement ratings (units)		117
**Safer Care for Older Persons [in residential] Environments**			
	Long-term care homes		45
	Participants		408
	**Surveys (participants; completed 4 times)**		
		Team level	159
		Individual level	478
		Leadership level	210
	Interviews or focus groups (participants)		331
	Observational data (sessions observed)		204

#### Case Study Data

We have data from 3 extensive ethnographic case studies completed in TREC phase 1 (2007-2012) [87,88]. We obtained 70 interviews and 22 sets of field notes from nurses, care aides, managers, allied health personnel, and family of residents from 3 LTC homes in Alberta, Saskatchewan, and Manitoba. In-person interviews (2008-2010) were semistructured. Ethnographic observations (2008-2009) were written as field notes by research associates. The purpose of the original ethnographic case studies was to explore how organizational context mediates staff use of evidence-based best practices in LTC homes [87].

#### Additional Variables Derived From TREC Data

Facilitation, implementation success, and improvement success are not directly available in our data, but we will derive the variables from our INFORM and SCOPE trial data. Many quality improvement interventions in health care settings target health care providers’ adoption of evidence-based best practices [89]. Our team has used survey data on staff adoption of best practices as a proxy for implementation success [56,85,90,91]. Specifically, we measured conceptual use of best practices [90,92] and instrumental use of best practices (applying best practice knowledge) [37,92]. In this study, we will draw on our earlier work to derive variables of implementation and improvement success using INFORM and SCOPE trial data [50,57]. We will rank the sites in each of our 2 trials on the basis of success, then derive a “success” variable, and experiment to find an optimal derivation.

We have published one conceptualization of facilitation [48]. Other data derivations are possible from examining role or process. We will rank facilitation effectiveness as we rank implementation and improvement success to derive a facilitation score by site.

### Analyses

#### Qualitative Data Analyses

We will examine qualitative data from our case studies and INFORM and SCOPE trials (process evaluation data). For the case study data, we will search for evidence on context elements that most strongly influence the association between facilitation and staff quality of work life, and between facilitation and improvements in resident outcomes. We will propose hypotheses for quantitative analysis that will contribute toward aims 2 and 3. For the trial data, we will look for evidence on the context elements that most strongly drive better trial intervention delivery, enactment, and receipt, and on improvement success of the trial. This will contribute toward our first aim of understanding implementation success. We will use ATLAS.ti software [93] to support data management and analysis and visualization of findings.

We will examine the data first with the lens of the PARIHS framework (eg, context and facilitation) and then augment with other key theoretical perspectives (eg, adaptive leadership and sense-making) [94,95]. We will use directed coding [96], guided by PARIHS concepts, and open coding [96] to capture concepts that are not part of PARIHS or that describe relevant staff and resident outcomes. Two coders will read and code each transcript and write a summary. Senior qualitative researchers on the coding team will review summaries. Codes will be added and refined, and summaries updated as needed.

Coders will then use the summaries to synthesize findings into a matrix to illustrate the concepts of interest and potential relationships among them. They will include sample quotes to exemplify the concepts. Matrices will be examined by senior team members and refinements made as needed. We will use the matrices to create network figures, using the network feature of ATLAS.ti software to depict relationships identified in the data. Networks will be reviewed by the full team and refined. Matrices and networks will be examined across cases to identify patterns and areas of contradiction. Questions from the full team will be explored by the coding team, who will look back at the original source data and examine the audit trail of the initial coding, summaries, matrices, and networks.

#### Quantitative Data Analyses

Our quantitative working group will analyze quantitative data from TREC surveys and RAI-MDS 2.0. We will be able to measure many constructs derived from qualitative analyses. TREC data are of high quality (few missing data and high response rates) and have been collected, cleaned, and processed through a rigorous process [97]. We will use a variety of statistical software packages for this work, such as SAS (SAS Institute), SPSS (IBM Corp), R (R Foundation for Statistical Computing), and STATA (StataCorp).

We will conduct basic descriptive and bivariate analyses, followed by more advanced statistical modeling using; for example, general estimating equations, hierarchical linear mixed models, and generalized linear mixed models. These enable us to adjust for complex nested structures of our data [26,38]. We will also explore the use of structural equation modeling and configurational modeling [98,99]. We will adjust our analyses as appropriate for different outcomes, including resident or care staff characteristics (depending on outcome), care unit characteristics, LTC home characteristics, and regional features depending on the addressed aim. We will begin with an approach that examines a standard set of context influences and assess it across staff and resident outcomes at unit and individual levels (aims 1 and 2). Based on these analyses, with the input of our qualitative team and panels, we will identify patterns and trends over time.

#### Mixed Methods Analysis

We will adopt convergent and sequential mixed methods models to obtain different but complementary data on the same problem for a more complete understanding [100]. In a convergent model, we will first analyze qualitative and quantitative data separately and independently, then integrate results in matrices, and interpret how the 2 sources of results converge or diverge [60,101]. In sequential modeling, results of qualitative analysis will inform the quantitative analysis and vice versa [60].

As an example, we will complement the PARIHS framework with other conceptual frameworks (eg, adaptive leadership and sense-making) [94,95,102] and form hypotheses related to our aims, including hypotheses on how context factors influence implementation outcomes. We will then test the hypotheses by analyzing both the quantitative and qualitative data and explore support or nonsupport of interrelationships. Next, we will focus on interactions among the context factors and interaction between context and facilitation based on our qualitative analyses, forming additional hypotheses. We will test these additional hypotheses with quantitative analysis. When unanticipated results emerge from the quantitative analysis, we will use qualitative analysis to explore processes and mechanisms explaining the results.

#### Refinement of Analyses

We will work iteratively with our stakeholder and expert panels to further inform and refine analyses. We will hold 2 virtual facilitated meetings with all panels (Figure 1). Before meetings, work groups will prepare summary reports of research findings and a focused set of guidelines and questions that are tailored to each panel. During meetings, the external expert panel will comment on summary reports and advise through scientific and theoretical lenses on early findings presented, most promising and important avenues to pursue, and areas not yet identified that our data would support for further exploration. The decision- and policy maker and LTC home manager panels will comment and advise on these same aspects through a lens of system management and utility of findings. The direct care staff panel will provide a frontline perspective on whether and how our findings could be useful to them and suggest areas we may not have considered. The citizen engagement panel will also comment and advise on whether and how our findings could be useful to them and suggest areas we may not have considered, from the perspective of findings important to their constituencies. This panel approach assists us with direction and focus, to keep our results both scientifically and practically relevant.

## Results

The results of the secondary data analysis are expected by the end of 2023.

## Discussion

This project responds to (1) calls to improve understanding of the mechanisms by which context and facilitation function, (2) criticisms that the aging field (like many) is rich in data but impoverished in theory, and (3) calls for optimized use of longitudinal data to advance the science of aging and appropriately inform policy makers on complex issues affecting aging populations [14,15,103,104].

We expect to advance the PARIHS framework by better describing the mechanisms by which context and facilitation influence implementation and improvement processes. We will identify specific factors of organizational context (such as slack time and space, culture, communication, and resources) and facilitation and understand how they interact and affect outcomes. We will elaborate on inner (local setting or organization) versus outer (health system, or policy or political infrastructure) context conditions.

We anticipate generating a stronger evidence base for large pragmatic implementation and quality improvement trials. An advantage of our study is the potential to generate both scientific and practical working knowledge through our integrated knowledge translation approach [105]. This maintains relevant to end users of the LTC system (residents and their family members) and the LTC care staff and managers who run the system and who are ultimately responsible for large-scale implementation of evidence-based choices.

## Appendix

Multimedia Appendix 1 Peer-review report by the Canadian Institutes of Health Research (CIHR) (Instituts de recherche en santé du Canada) - Knowledge Translation Research (Recherche sur l'application des connaissances) (Canada).

## Abbreviations

ACT: Alberta Context Tool
HRDR: Health Research Data Repository
INFORM: Improving Nursing home care through Feedback On perfoRMance
LTC: Long-term care
PARIHS: Promoting Action on Research Implementation in Health Services
RAI-MDS: Resident Assessment Instrument – Minimum Dataset
SCOPE: Safer Care for Older Persons [in residential] Environments
TREC: Translating Research in Elder Care

## Data Availability

The data used for this article are housed in the secure and confidential Health Research Data Repository (HRDR) in the Faculty of Nursing at the University of Alberta, in accordance with the health privacy legislation of participating Translating Research in Elder Care (TREC) jurisdictions. These health privacy legislations and the ethics approvals covering TREC data do not allow public sharing or removal of completely disaggregated data (resident-level records) from the HRDR, even if deidentified. The data were provided under specific data sharing agreements only for approved use by TREC within the HRDR. Where necessary, access to the HRDR to review the original source data may be granted to those who meet prespecified criteria for confidential access, available at request from the TREC data unit manager, with the consent of the original data providers and the required privacy and ethical review bodies. Statistical and anonymous aggregate data, the full data set creation plan, and underlying analytic code associated with this paper are available from the authors upon request, understanding that the programs may rely on coding templates or macros that are unique to TREC.
