# Supplementary material for: The Influence of Context on Implementation and Improvement: Protocol for a Mixed Methods, Secondary Analyses Study
Source: JMIR Res Protoc. 2022 Sep 15;11(9):e40611. doi: 10.2196/40611 (PMC9523530; doi:10.2196/40611)
Supplement: Multimedia Appendix 1 [file resprot_v11i9e40611_app1.pdf]

**Review Type / Type d'évaluation:**	Reviewer 1 / Évaluateur 1
**Name of Applicant / Nom du chercheur:**	Estabrooks, Carole Anne
**Application No. / Numéro de demande:**	420645
**Agency / Agence:**	CIHR/IRSC
**Competition / Concours:**	Project Grant/Subvention Projet
**Committee / Comité:**	Knowledge Translation Research/Recherche sur l'application des connaissances
**Title / Titre:**	The Influence of Context on Implementation and Improvement

Adjudication Criteria/Critères de sélection

Significance and Impact of the Research/Importance et impact de la recherche: 4.9

Approaches and Methods/Approches et méthodes: 4.9

Expertise, Experience and Resources/Expertise, expérience et ressources: 4.9

Top/Bottom Selection/Groupe supérieur/inférieur

◉ **Top/Groupe supérieur**
○ **Bottom/Groupe inférieur**

**Review Type / Type d'évaluation:**	Reviewer 1 / Évaluateur 1
**Name of Applicant / Nom du chercheur:**	Estabrooks, Carole Anne
**Application No. / Numéro de demande:**	420645
**Agency / Agence:**	CIHR/IRSC
**Competition / Concours:**	Project Grant/Subvention Projet
**Committee / Comité:**	Knowledge Translation Research/Recherche sur l'application des connaissances
**Title / Titre:**	The Influence of Context on Implementation and Improvement

Summary of Application/Résumé de la demande:

The proposed work centres on context and facilitation within long term care settings. Specifically, the authors are interested in how context influences the success of implementation and QI initiatives, and how contextual conditions within which facilitation affects staff worklife and resident outcomes. This project is a secondary analysis of a huge quantitative and qualitative 12 year dataset held by the research team. Using a mixed methods design, the qualitative data will be mined for hypothesis related to the project aims. The quantitative dataset will be used to build models reflecting the hypotheses. Panels of users (managers and health system leaders), researchers, residents/family and an expert panel will continuously be engaged to direct the research. Anticipated outcomes and outputs are described.

**Review Type / Type d'évaluation:**	Reviewer 1 / Évaluateur 1
**Name of Applicant / Nom du chercheur:**	Estabrooks, Carole Anne
**Application No. / Numéro de demande:**	420645
**Agency / Agence:**	CIHR/IRSC
**Competition / Concours:**	Project Grant/Subvention Projet
**Committee / Comité:**	Knowledge Translation Research/Recherche sur l'application des connaissances
**Title / Titre:**	The Influence of Context on Implementation and Improvement

Strengths and Weaknesses/Forces et faiblesses:

Strength

The team is extraordinary. The PI has cultivated relationships with relevant knowledge users in Alberta, Nova Scotia, British Columbia and Manitoba; the relationships with these individuals and/or their organizations have been on-going for several years. These individuals and their organizations are important because they have the influence to implement any of the findings arising from this work. Further, the PI has brought together a strong mix of senior researchers, junior researchers and trainees. To complement this expertise, the research team has pulled together five important panels as part of the research process. These panels, composed of researchers, end users at the system and faculty level, resident/family members and an external expert panel, will provide input about relevance, feasibility and methodological rigour. Both Canadian and international investigators and panel members will ensure the work will have reach beyond the national setting.

The approach to this 42 month project is stellar. An extensive literature review is presented. Clear goals, aims and outcomes align with the proposed research activities. The work is theoretically informed by the PARIHS framework, complex adaptive systems, organizational learning and goal setting theory. The analysis is based on robust data that have been collected over 12 years from different types of studies (trials, case studies, pilot studies, cohort study) using psychometrically sound instruments. Qualitative data have also been collected. This proposed work will use the qualitative data to generate hypothesis which will be further explored using the quantitative data. Figure 1 is particularly helpful for depicting how the team and panels will intersect with the research cycles.

The importance of this dedicated focus on the long term care sector cannot be underestimated. As the authors point out, this work is significant given the ageing population. Not only will long term care homes experience an increase in numbers of residents, but they will enter with more complex co-morbidities than ever before. This work is part of a program of research that is comprehensive (quality of staff worklife, resident outcomes, quality of care), longitudinal, informed by external realities (practice and policy) and robust. The impact of this work is assured.

Weakness

The authors might have provided a sentence or two to justify their decision to use the qualitative data for hypothesis generation rather than using the data in other ways within the mixed methods design.

The authors note that the qualitative data analysis might result in hypothesis around variables which weren't captured by the team (p. 6). It wasn't clear how the team was going to address this problem. Would these hypotheses be taken up in future primary studies?

**Review Type / Type d'évaluation:**	Reviewer 1 / Évaluateur 1
**Name of Applicant / Nom du chercheur:**	Estabrooks, Carole Anne
**Application No. / Numéro de demande:**	420645
**Agency / Agence:**	CIHR/IRSC
**Competition / Concours:**	Project Grant/Subvention Projet
**Committee / Comité:**	Knowledge Translation Research/Recherche sur l'application des connaissances
**Title / Titre:**	The Influence of Context on Implementation and Improvement

Budget Recommendation/Recommandation budgétaire:

Accepted, as described

Please indicate your appraisal of the integration of sex as a biological variable as a strength, weakness, or not applicable to the proposal./Prière de sélectionner une option pour donner votre évaluation de l'intégration du sexe comme variable biologique en tant que point fort ou point faible de la proposition, ou en tant qu'élément non applicable à la proposition.

- (●) **Strength/Point fort**
- ( ) **Weakness/Point faible**
- ( ) **Not applicable/Non applicable**

Please indicate your appraisal of the integration of gender as a socio-cultural determinant of health as a strength, weakness, or not applicable to the proposal./Prière de sélectionner une option pour donner votre évaluation de l'intégration du genre comme déterminant socioculturel de la santé en tant que point fort ou point faible de la proposition, ou en tant qu'élément non applicable à la proposition.

- ( ) **Strength/Point fort**
- (●) **Weakness/Point faible**
- ( ) **Not applicable/Non applicable**

**Review Type / Type d'évaluation:**	Reviewer 1 / Évaluateur 1
**Name of Applicant / Nom du chercheur:**	Estabrooks, Carole Anne
**Application No. / Numéro de demande:**	420645
**Agency / Agence:**	CIHR/IRSC
**Competition / Concours:**	Project Grant/Subvention Projet
**Committee / Comité:**	Knowledge Translation Research/Recherche sur l'application des connaissances
**Title / Titre:**	The Influence of Context on Implementation and Improvement

Sex and/or Gender Considerations/Notions de sexe et/ou de genre:

The team will explore sex and gender in their analysis. The key consideration here is that their existing dataset includes information on sex, allowing them to investigate the sex composition of teams on implementation success, for example.

**Review Type / Type d'évaluation:**	Reviewer 2 / Évaluateur 2
**Name of Applicant / Nom du chercheur:**	Estabrooks, Carole Anne
**Application No. / Numéro de demande:**	420645
**Agency / Agence:**	CIHR/IRSC
**Competition / Concours:**	Project Grant/Subvention Projet
**Committee / Comité:**	Knowledge Translation Research/Recherche sur l'application des connaissances
**Title / Titre:**	The Influence of Context on Implementation and Improvement

Adjudication Criteria/Critères de sélection

Significance and Impact of the Research/Importance et impact de la recherche: 4.5

Approaches and Methods/Approches et méthodes: 4.6

Expertise, Experience and Resources/Expertise, expérience et ressources: 4.8

Top/Bottom Selection/Groupe supérieur/inférieur

- (●) **Top/Groupe supérieur**
- ( ) **Bottom/Groupe inférieur**

**Review Type / Type d'évaluation:**	Reviewer 2 / Évaluateur 2
**Name of Applicant / Nom du chercheur:**	Estabrooks, Carole Anne
**Application No. / Numéro de demande:**	420645
**Agency / Agence:**	CIHR/IRSC
**Competition / Concours:**	Project Grant/Subvention Projet
**Committee / Comité:**	Knowledge Translation Research/Recherche sur l'application des connaissances
**Title / Titre:**	The Influence of Context on Implementation and Improvement

Summary of Application/Résumé de la demande:

This IKT project will explore context and facilitation related to uptake of research, organizational performance, and health outcomes. The project is structured as a sequential exploratory and confirmatory mixed-methods design, taking place over 42 months. At the end of the project, the ways in which context can influence success of implementation of quality improve initiatives, and the conditions under which facilitation affects staff quality of worklife outcomes as well as resident outcomes.

**Review Type / Type d'évaluation:**	Reviewer 2 / Évaluateur 2
**Name of Applicant / Nom du chercheur:**	Estabrooks, Carole Anne
**Application No. / Numéro de demande:**	420645
**Agency / Agence:**	CIHR/IRSC
**Competition / Concours:**	Project Grant/Subvention Projet
**Committee / Comité:**	Knowledge Translation Research/Recherche sur l'application des connaissances
**Title / Titre:**	The Influence of Context on Implementation and Improvement

Strengths and Weaknesses/Forces et faiblesses:

Excellent team with excellent track records; the impression is that this research team runs like a machine. Fascinating and relevant topic. Very thorough and compelling background section. Well articulated design--a stellar example of mixed methods research. Fully engaged, authentic relationships, meaningful partnerships with a wide and active network of stakeholders. The TREC work is already having widespread impacts geographically.

**Review Type / Type d'évaluation:**	Reviewer 2 / Évaluateur 2
**Name of Applicant / Nom du chercheur:**	Estabrooks, Carole Anne
**Application No. / Numéro de demande:**	420645
**Agency / Agence:**	CIHR/IRSC
**Competition / Concours:**	Project Grant/Subvention Projet
**Committee / Comité:**	Knowledge Translation Research/Recherche sur l'application des connaissances
**Title / Titre:**	The Influence of Context on Implementation and Improvement

Budget Recommendation/Recommandation budgétaire:

Please indicate your appraisal of the integration of sex as a biological variable as a strength, weakness, or not applicable to the proposal./Prière de sélectionner une option pour donner votre évaluation de l'intégration du sexe comme variable biologique en tant que point fort ou point faible de la proposition, ou en tant qu'élément non applicable à la proposition.

- (●) **Strength/Point fort**
- ( ) **Weakness/Point faible**
- ( ) **Not applicable/Non applicable**

Please indicate your appraisal of the integration of gender as a socio-cultural determinant of health as a strength, weakness, or not applicable to the proposal./Prière de sélectionner une option pour donner votre évaluation de l'intégration du genre comme déterminant socioculturel de la santé en tant que point fort ou point faible de la proposition, ou en tant qu'élément non applicable à la proposition.

- (●) **Strength/Point fort**
- ( ) **Weakness/Point faible**
- ( ) **Not applicable/Non applicable**

**Review Type / Type d'évaluation:**	Reviewer 2 / Évaluateur 2
**Name of Applicant / Nom du chercheur:**	Estabrooks, Carole Anne
**Application No. / Numéro de demande:**	420645
**Agency / Agence:**	CIHR/IRSC
**Competition / Concours:**	Project Grant/Subvention Projet
**Committee / Comité:**	Knowledge Translation Research/Recherche sur l'application des connaissances
**Title / Titre:**	The Influence of Context on Implementation and Improvement

Sex and/or Gender Considerations/Notions de sexe et/ou de genre:

**Review Type / Type d'évaluation:**	Reviewer 3 / Évaluateur 3
**Name of Applicant / Nom du chercheur:**	Estabrooks, Carole Anne
**Application No. / Numéro de demande:**	420645
**Agency / Agence:**	CIHR/IRSC
**Competition / Concours:**	Project Grant/Subvention Projet
**Committee / Comité:**	Knowledge Translation Research/Recherche sur l'application des connaissances
**Title / Titre:**	The Influence of Context on Implementation and Improvement

Adjudication Criteria/Critères de sélection

Significance and Impact of the Research/Importance et impact de la recherche: 4.2

Approaches and Methods/Approches et méthodes: 3.7

Expertise, Experience and Resources/Expertise, expérience et ressources: 4.8

Top/Bottom Selection/Groupe supérieur/inférieur

- ◉ **Top/Groupe supérieur**
- ○ **Bottom/Groupe inférieur**

**Review Type / Type d'évaluation:**	Reviewer 3 / Évaluateur 3
**Name of Applicant / Nom du chercheur:**	Estabrooks, Carole Anne
**Application No. / Numéro de demande:**	420645
**Agency / Agence:**	CIHR/IRSC
**Competition / Concours:**	Project Grant/Subvention Projet
**Committee / Comité:**	Knowledge Translation Research/Recherche sur l'application des connaissances
**Title / Titre:**	The Influence of Context on Implementation and Improvement

Summary of Application/Résumé de la demande:

This project focuses on two critical factors that influence the success of implementation and improvement efforts, as well as, resident and care staff outcomes. These are: a) modifiable features of organizational context, and b) modifiable aspects of facilitation roles and processes (argued to improve uptake of research, organizational performance and health outcomes).
Our specific aims are to:
1. Identify how context influences success of implementation and of quality improvement initiatives (focusing on our successful trials).
2. Identify and map context conditions in which facilitation affects staff quality of worklife outcomes (e.g., burnout, satisfaction, work engagement).
3. Identify and map context conditions in which facilitation affects resident outcomes (e.g., care quality).
A mixed methods project involving secondary data analysis is proposed. TREC data from multiple sources, in multiple forms, collected from 2007–2019 are available. Data holdings include quantitative data (continuously collected administrative data using the RAI-MDS 2.0 instrument, 5 waves of primary surveys from multiple care provider groups in the residential long term care sector (15,000 cases to date)) and extensive qualitative data (case studies, extensive process evaluations from 2 large trials). We will use a variety of analytic methods that account for our data clustering (quantitative) and a variety of qualitative techniques commonly used in mixed methods research (e.g., constant comparison).

Expected outcomes include: 1)New knowledge about how context and facilitation influence the success of implementation and improvement successes, and about how they interact to do this; 2) advances to the PARIHS framework specifically and to the broader literature on the mechanisms by which context and facilitation influence implementation and improvement processes; 3) a stronger evidence base for designing interventions in large pragmatic implementation and quality improvement trials; 4) advances in the utility and practical application of this work in complex health systems.

Strengths and Weaknesses/Forces et faiblesses:

25% Significance and Impact of the Research:

Strengths

•Key terms, concept and facilitation, defined.

•iKT approach taken, longstanding partnership with KUs from LTC setting.

•Evidence supporting the link between context and facilitation and 3 categories of outcomes: success of improvement initiatives, quality of care for LTC residents, and quality of worklife for paid staff, provide a solid rationale for the proposed research.

•Strong rationale for targeting the LTC setting given that residents experience a high rate of burdensome symptoms and inappropriate care at end of life, and unregulated care aides are at high risk for negative mental health outcomes.

•An overview of the PARIHS framework is provided and its weaknesses identified. Theories and frameworks used by the research team to complement the guidance provided by PARIHS are listed.

•Comprehensive KT plan.

Weaknesses

•A rationale for selecting the PARIHS framework over other frameworks for examining context and facilitation would strengthen the rationale for this research.

•A brief description of the data that are available for the proposed research in the background section would enable the reader to better understand the proposed project goals and aims which are somewhat non-specific. For example what types of quality improvement initiatives are the focus of specific research aim 1? In aim 2, does “staff” refer to all staff in the LTC setting? Or just unregulated care aides? Aim 3 would be improved by specifying the outcomes of interest.

•Presumably all project goals and aims relate the LTC setting but this is not stated.

50% Approaches and Methods:

Strengths

•Feasibility supported by availability of data in this secondary data analysis.

•Data are from 94 LTC facilities across 4 western provinces which support the external validity of the results.

•Table 1 indicates that extensive data are available which suggests the project will yield robust results.

•Central housing of the data in the Health Research Data Repository (University of Alberta Faculty of Nursing) facilitates the proposed research.

•The proposed statistical analysis accounts for the clustered nature of the data at multiple levels.

•Use of panels of internal and external stakeholders, including a citizen engagement group of people with dementia, caregivers of LTC residents, and international scientists, throughout the research provides strong evidence of the iKT approach and will help ensure the relevance of the research.

•Some potential risks and mitigation strategies are identified.

Weaknesses

•The proposed methods are difficult to follow. A high level overview of the TREC data available is first provided, and limited detail as to how the outcomes (staff quality of worklife, quality of resident care) were measured is not given until page 8. A comprehensive description of the quantitative data (ie constructs/variables), the quantitative measurement tools used, and the measurement properties of those tools is not provided making it difficult to judge the quality of the data.
•No description is given of the aims of the interviews that produced the qualitative data that would enable a judgment of the feasibility of the qualitative data matching onto the quantitative data as proposed in figure 1. The RAI-MDS 2.0 is the only measure named in the methods section.
•A description of variables and how they were measured is needed prior to the quantitative data analysis section to evaluate the appropriateness of the analysis proposed.
•The “panels” mentioned in Figure 1 on page 5 are not clarified until page 7 making the proposed methodology difficult to follow.

25% Expertise, Experience and Resources:
Strengths
•NPA Estabrooks holds a Tier 1 Canada Research Chair and has strong expertise and experience related to the focus of the proposed research (examining the influence of context in LTC settings, use of PARIHS) as supported by numerous publications and grants.
•The team has extensive experience with the proposed quantitative statistical analyses proposed.
•This team has depth and breadth in disciplinary composition, the methodological expertise, and a rich history of partner engagement. The researchers include senior, mid-career and junior investigators and will enable the introduction of postdoctoral trainees into the project, and other trainees as research assistants.
•The team has senior decision making partners and end users and the methodological approach reflects extensive engagement of policy makers, system managers, residents, their caregivers, and an external panel of substantive experts.

Weaknesses
•Whitney Berta is mentioned as a co-applicant but she is not listed under project details.

**Review Type / Type d'évaluation:**	Reviewer 3 / Évaluateur 3
**Name of Applicant / Nom du chercheur:**	Estabrooks, Carole Anne
**Application No. / Numéro de demande:**	420645
**Agency / Agence:**	CIHR/IRSC
**Competition / Concours:**	Project Grant/Subvention Projet
**Committee / Comité:**	Knowledge Translation Research/Recherche sur l'application des connaissances
**Title / Titre:**	The Influence of Context on Implementation and Improvement

Budget Recommendation/Recommandation budgétaire:

Accepted as presented?Yes

Reduced by a specific dollar amount (in total, not by year):0

The scoping review proposed in phase 1 of the research proposal has begun in the last year so it is unclear whether funds are being requested for this activity.

**Review Type / Type d'évaluation:**	Reviewer 3 / Évaluateur 3
**Name of Applicant / Nom du chercheur:**	Estabrooks, Carole Anne
**Application No. / Numéro de demande:**	420645
**Agency / Agence:**	CIHR/IRSC
**Competition / Concours:**	Project Grant/Subvention Projet
**Committee / Comité:**	Knowledge Translation Research/Recherche sur l'application des connaissances
**Title / Titre:**	The Influence of Context on Implementation and Improvement

Please indicate your appraisal of the integration of sex as a biological variable as a strength, weakness, or not applicable to the proposal./Prière de sélectionner une option pour donner votre évaluation de l'intégration du sexe comme variable biologique en tant que point fort ou point faible de la proposition, ou en tant qu'élément non applicable à la proposition.

- (●) **Strength/Point fort**
- ( ) **Weakness/Point faible**
- ( ) **Not applicable/Non applicable**

Please indicate your appraisal of the integration of gender as a socio-cultural determinant of health as a strength, weakness, or not applicable to the proposal./Prière de sélectionner une option pour donner votre évaluation de l'intégration du genre comme déterminant socioculturel de la santé en tant que point fort ou point faible de la proposition, ou en tant qu'élément non applicable à la proposition.

- (●) **Strength/Point fort**
- ( ) **Weakness/Point faible**
- ( ) **Not applicable/Non applicable**

**Review Type / Type d'évaluation:**	Reviewer 3 / Évaluateur 3
**Name of Applicant / Nom du chercheur:**	Estabrooks, Carole Anne
**Application No. / Numéro de demande:**	420645
**Agency / Agence:**	CIHR/IRSC
**Competition / Concours:**	Project Grant/Subvention Projet
**Committee / Comité:**	Knowledge Translation Research/Recherche sur l'application des connaissances
**Title / Titre:**	The Influence of Context on Implementation and Improvement

Sex and/or Gender Considerations/Notions de sexe et/ou de genre:

The research team plans collect quantitative data on sex and qualitative data collection will include information about sex and gender. The team will explore the intersection of sex and gender in their analyses.

Review Type/Type d'évaluation: SO Notes /Notes de l'agent scientifique
Name of Applicant/Nom du chercheur: Estabrooks, Carole Anne
Application No./Numéro de demande: 420645
Agency/Agence: CIHR/IRSC
Competition/Concours: 2019-03-06 Project Grant/Subvention Projet
Committee/Comité: Knowledge Translation Research/Recherche sur l'application des connaissances
Title/Titre: The Influence of Context on Implementation and Improvement

Assessment/Évaluation:

SO Notes begin here:
````````````````````````````````````````````````````````````````````````````````````````````````````````

**Strengths**

- This is an excellent team with many years of accumulated experience of working together effectively in this area. Its large cohort of applicants includes an impressive mix of skills and levels of experience as well as both Canadian and international affiliations.
- The team has accumulated a very large high-quality dataset derived from a range of study types that it intends to mine for this project.
- It has also developed and sustained an impressive network of solid relationships with well-chosen stakeholders and partners that will be very important in the planned work.
- The issues to be studied are important ones both for the specific domain of elder care and for a wider array of topics and for KT work in general.
- The research program proposed involves the use of multiple panels with a variety of members including external experts, stakeholders and patients
- The proposed work is firmly anchored in a well-established methodology framework (PARIHS) and grounded in a carefully performed review of the appropriate literature.
- The applicants have paid appropriate attention to sex/gender issues and will be able to assess the impact of sex/gender on effective implementation.

**Weaknesses**

- The use of the PARIHS framework rather than some other is not justified.
- Their dataset includes both qualitative and quantitative data which is an asset but the alignment of the two types of data is not fully addressed. For example, how can the qualitative data be used to fill in gaps on issues on which the quantitative data are silent or incomplete? Providing some details about the kinds of questions asked in the qualitative work would have helped reviewers judge how the qualitative data will match up with the quantitative.
- No details about the dataset are provided except in an appendix and this makes it hard to evaluate the plan for data analysis.
- Not clear what categories of staff they will be considering.

************************************************************************************
````````````````````````````````````````````````````````````````````````````````````````````````````````

Review Type/Type d'évaluation: SO Notes /Notes de l'agent scientifique
Name of Applicant/Nom du chercheur: Estabrooks, Carole Anne
Application No./Numéro de demande: 420645
Agency/Agence: CIHR/IRSC
Competition/Concours: 2019-03-06 Project Grant/Subvention Projet
Committee/Comité: Knowledge Translation Research/Recherche sur l'application des connaissances
Title/Titre: The Influence of Context on Implementation and Improvement

---

Assessment/Évaluation:

Note: The final rating of the application, provided in the Notice of Decision (NOD), is the averaged rating of the peer review committee members following the discussion of the application during the committee meeting, and therefore may differ from the ratings provided by the assigned reviewers in their respective reviews.

Remarque : La cote définitive de la demande, qui apparaît dans l’avis de décision, représente la moyenne des cotes accordées par les membres du comité d’ évaluation par les pairs après avoir débattu de la demande à la réunion du comité. Elle peut donc différer de celle donnée par les évaluateurs dans leur évaluation respective.
````````````````````````````````````````````````````````````````````````````````````````````````````````````````````````

***SO Notes end here.***
````````````````````````````````````````````````````````````````````````````````````````````````````````````````````````
